# The hormonal environment and estrogen receptor signaling alters *Chlamydia muridarum* infection *in vivo*

**DOI:** 10.3389/fcimb.2022.939944

**Published:** 2022-12-27

**Authors:** Amy Gravitte, Jennifer Kintner, Stacy Brown, Allison Cobble, Benjamin Kennard, Jennifer V. Hall

**Affiliations:** ^1^ Department of Biomedical Sciences, Quillen College of Medicine, East Tennessee State University, Johnson City, TN, United States; ^2^ Center of Excellence for Inflammation, Infection Disease, and Immunity, East Tennessee State University, Johnson City, TN, United States; ^3^ Department of Pharmaceutical Sciences, Bill Gatton College of Pharmacy, East Tennessee State University, Johnson City, TN, United States

**Keywords:** chlamydia, estrogen receptor, ovariectomized mouse model, long-term hormone delivery, estrogen receptor knockout mice

## Abstract

Genital *Chlamydia* is the most common bacterial sexually transmitted infection in the United States and worldwide. Previous studies indicate that the progression of chlamydial infection is influenced by various factors, including the female sex hormones estrogen and progesterone. Sex hormone levels naturally fluctuate in women throughout their menstrual cycle. Varying concentrations of estrogen and progesterone may impact the progression of chlamydial infection and the host’s immune response to *Chlamydia*. Estrogen signals through estrogen receptors (ERs), ERα and ERβ. These receptors are similar in structure and function, but are differentially expressed in tissues throughout the body, including the genital tract and on cells of the immune system. In this study, we used ovariectomized (OVT) BALB/c mice to investigate the impact of long-term administration of physiologically relevant concentrations of estrogen (E2), progesterone (P4), or a combination of E2/P4 on the progression of and immune response to *C. muridarum* infection. Additionally, we used ERα and ERβ knockout C57/BL6 mice to determine the how ERs affect chlamydial infection and the resulting immune response. Estrogen exposure prevented *C. muridarum* infection in vaginally infected OVT mice exposed to E2 alone or in combination with P4, while OVT or Sham mice exposed to hormone free, P4 or depo-medroxyprogesterone acetate shed similar amounts of chlamydiae. The hormonal environment also altered T cell recruitment and IFNϵ production the genital tracts of infected OVT and Sham mice on day 10 post infection. The absence of ERα, but not ERβ, in ER knockout mouse strains significantly changed the timing of *C. muridarum* infection. ERαKO mice shed significantly more chlamydiae at day 3 post infection and resolved the infection faster than WT or ERβKO animals. At day 9 post infection, flow cytometry showed that ERαKO mice had more T cells present and targeted RNA sequencing revealed increased expression of *CD4* and *FOXP3*, suggesting that ERαKO mice had increased numbers of regulatory T cells compared to ERβKO and WT mice. Mock and chlamydia-infected ERαKO mice also expressed more IFNϵ early during infection. Overall, the data from these studies indicate that sex hormones and their receptors, particularly ERα and ERβ, differentially affect *C. muridarum* infection in murine models of infection.

## 1 Introduction

*Chlamydiae* are obligate intracellular bacteria that cause mucosal infections in vertebrates including humans, other mammals, and birds. Primary sites of infection include the conjunctiva, the respiratory tract, and the genital tract (Bachmann et al., 2014). Genital *Chlamydia* infections in humans are caused by *Chlamydia trachomatis*, the most reported bacterial sexually transmitted pathogen in the United States (US) and worldwide. Reported cases of genital *Chlamydia* infections are rising with a 2.9% increase in reported cases in the US from 2017-2018 (Kreisel et al., 2021). *C. trachomatis* exists in two developmental forms as it progresses through its biphasic life cycle: infectious, non-replicative elementary bodies (EB) and non-infectious, replicative reticulate bodies (RB) (Moulder, 1991). As chlamydial infection progresses in females, it ascends the genital tract, invading the endometrium and fallopian tubes. Left untreated, this can result in severe sequelae such as pelvic inflammatory disease, ectopic pregnancy, and irreversible tubal infertility (Paavonen, 1999).

Estrogen is a female sex steroid hormone that primarily signals through two estrogen receptors (ERs): estrogen receptor alpha (ESR1, ERα) and estrogen receptor beta (ESR1, ERβ), as well as a G protein-coupled estrogen receptor, GPER-1 (formerly known as GPR30) (Eyster, 2016). Cytoplasmic ERs alter gene transcription *via* two mechanisms when activated by estrogen. In the classical mechanism, estrogen binds the ER causing translocation to the nucleus and activation of gene transcription *via* direct binding to DNA (Katzenellenbogen et al., 2000). In the tethered mechanism, estrogen-stimulated ERs form complexes with DNA-bound transcription factors to modulate the activity of the transcription factors (Jakacka et al., 2001; Heldring et al., 2011). In addition to these nuclear signaling mechanisms, classical ERs (ERα and ERβ) act in a non-nuclear fashion by stimulating protein kinases such as c-Src and mitogen-activated protein kinase (MEK) (Pedram et al., 2006; Madak-Erdogan et al., 2008). Membrane ERs and nuclear ERs are derived from the same mRNA transcript (Razandi et al., 1999; Chambliss et al., 2002). The amount and identity of ERs (α or β) found at the plasma membrane is dependent on cell type, but it is estimated that 5-10% of ERs are located in the plasma membrane (Levin, 2009). Estrogen signaling *via* membrane ERs triggers cyclic adenosine monophosphate (cAMP) and extracellular signal-regulated kinase (ERK) activity to stimulate non-genomic effects (Razandi et al., 2000; Song et al., 2002; Pedram et al., 2006). GPER-1 is reported to mediate membrane-associated estrogen signaling (Prossnitz et al., 2008). However, some reports contradict this observation, noting that the absence of GPER-1 does not affect estrogen signaling and GPER-1 does not compensate for estrogen signaling in the absence of ERs (Pedram et al., 2006).

As human females progress through a typical 28-day menstrual cycle (Caligioni, 2009), estrogen and progesterone levels naturally rise and fall. As estrogen levels rise during the proliferative phase of the menstrual cycle, both the endometrium and vaginal epithelium thickens and cervical mucus increases. Following ovulation, estrogen levels fall and progesterone becomes the predominant hormone in the secretory phase as the uterus prepares for either implantation or menstruation. At the end of the secretory phase, progesterone levels decline, causing the endometrial epithelial lining to be shed during menses. Varying levels of estrogen affect the expression of cytokines, chemokines, pattern recognition receptors, and other immune components (Wira et al., 2015). The estrous cycle in mice is similar to the reproductive cycle in humans, though it is much shorter at 4-5 days in length (Caligioni, 2009). During the murine estrous cycle, estrogen concentrations rise during proestrus, remain elevated during estrus, fall during metestrus, and begin to increase again during diestrus (Caligioni, 2009). The concentration of progesterone in mice is low during proestrus and estrus, begins to rise during metestrus, and peaks during diestrus (Walmer et al., 1992).

Several studies indicate that estrogen and its receptors are involved in the establishment and progression of chlamydial infection. The effects of estrogen on chlamydial disease are likely multifactorial in nature, involving the functions of multiple cell types including epithelial, stromal and immune cells. Estrogen exposure has been shown to affect the expression of toll-like receptors (TLR) as well as chemokine and cytokine production (Agrawal et al., 2013; Fung et al., 2013). EB interact with the surface of epithelial host cells through protein disulfide isomerase, a component of the membrane estrogen receptor complex (Davis et al., 2002). Previously, our laboratory demonstrated that 17-β estradiol treatment prior to infection with *C. trachomatis* resulted in an increase in chlamydial inclusion development and infectious EB production in the estrogen-responsive endometrial cell line, Ishikawa, co-cultured with the stromal cell line SHT-290. Additionally, antibody blockage of ERα and/or ERβ decreased chlamydial infectivity in both Ishikawa and Hec-1b cell lines (J. V. Hall et al., 2011). Our prior data, along with the understanding that estrogen/ER signaling modulates the immune response suggests that ERα and/or ERβ are involved in the host’s response chlamydial infection. While tissue culture models are useful for examining the effects of hormone-induced, cell communication on chlamydial infection, it does not recapitulate the *in vivo* setting with a functioning immune system.

Previous work has shown that sex hormones alter the progression of chlamydial infections *in vivo*. However, the hormone concentration, timing and route of administration vary in previous studies. Most of these models neither reported the serum concentrations of hormones after delivery to ensure that physiologically relevant conditions were maintained nor examined the effects of long-term hormone exposure on chlamydial development *in vivo.* Additionally, previous studies did not evaluate the specific contributions of ERα and/or ERβ signaling on chlamydial infection *in vivo*. Thus, the current study aims to elucidate the complex network of interactions between female sex hormones, estrogen receptor activity, chlamydial infection, and the immune response using two *C. muridarum* infection models.

## 2 Materials/methods

### 2.1 *Chlamydia* and tissue culture cell lines

*Chlamydia muridarum* strain *Nigg* was cultured in HeLa 229 cells grown on Cytodex microcarrier beads. EB were harvested by centrifugation and stored in 2SPG [0.02 M phosphate buffer, 0.2 M sucrose, 5mM glutamine (pH 7.2)] at -80°C. The human cervical cell line HeLa 229 was maintained in Minimal Essential Medium (Gibco) with 10% fetal calf serum (FBS) and 0.01% gentamycin.

### 2.2 Mouse strains

To investigate the effects of estrogen and progesterone on *C. muridarum* infection in an experimentally controllable animal model, ovariectomized (OVT) or sham-OVT female BALB/c mice were ordered from the Jackson Laboratory (Bar Harbor, ME). Surgery was performed at 5 weeks of age, and mice were delivered at 6 weeks of age. Upon arrival, vaginal washes were performed with 20ul 1X PBS to ensure the mice were not experiencing an estrous cycle (**Supplemental Figure 1**). Four genotypes of 6–8-week-old C57/BL6 mice were ordered from the Jackson Laboratory (Bar Harbor, ME): B6N(Cg)-*Esr1^tm4.2Ksk^/*J (ERαKO), C57BL/6NJ (ERαWT), B6.129P2-*Esr2^tm1Unc^*/J (ERβKO) and C57BL/6J (ERβWT) to investigate the specific effects of ERα and ERβ signaling on *C. muridarum* infection. All mice were housed 4-5 per cage in a facility with a 12:12 light/dark cycle and provided with food and water *ad libitum.* All animal research was conducted according to our vertebrate animal protocol as approved by the ETSU University Committee on Animal Care, accredited through the Assessment and Accreditation of Laboratory Animal Care.

### 2.3 Making and insertion of silastic capsules

Mice were exposed to estrogen or progesterone *via* a surgically implanted silastic capsule as described by Ingberg, et al. (Ingberg et al., 2012) and Strom, et al. (Ström et al., 2012). Solutions of 36 ug/ml 17-β estradiol (E2), 50 mg/ml progesterone (P4), and 36 ug/ml 17-β estradiol + 50 mg/ml progesterone (E2/P4) were prepared in sesame oil (SO). Two cm lengths of silastic tubing were filled with the appropriate solution (E2, P4, E2/P4, or SO alone) and the ends were plugged with 3mm wooden applicator sticks. Capsules were placed in the remaining hormone solution and left on a rocker at 37°C for 2 days to equilibrate.

OVT or sham-OVT mice were anesthetized with isofluorane. A 2x2 cm area between the mouse’s shoulders was shaved and cleaned with iodine. An incision was made, and a subcutaneous pocket was bluntly dissected to make space for the capsule. The capsule was inserted, and the incision was closed with 1-2 staples. Mice were monitored for two days post-surgery to confirm the incision was healing and the capsule was retained. One-week post-surgery, the staples were removed under isofluorane anesthesia.

### 2.4 *C. muridarum* infection and titer of progeny EB

Mice were vaginally infected with 500 IFU of *C. muridarum* in 2SPG eight days post hormone capsule insertion (OVT mice) or one week after depo-medroxyprogesterone acetate (DMPA, 2mg/kg) treatment (non-OVT mice). Every three days post infection (pi) mice were vaginally swabbed with a calcium alginate tipped swab, which was stored at -80°C. Hela 229 monolayers were infected in duplicate with a dilution of the mouse vaginal swab samples by centrifugation. Infected monolayers were incubated 24 hours before methanol fixation. Inclusions in the fixed samples were stained with anti-LPS stain (Biorad) and viewed using a Ziess Axiovert fluorescent microscope. The number of inclusion forming units (IFU) were counted on each coverslip and IFU per swab sample was calculated to create a 21 day EB shed curve for each mouse (Campbell et al., 2012).

### 2.5 Serum collection for hormone measurements

Blood was collected *via* cheek bleed into a 1.5 mL tube and left at room temperature for 30 minutes, then centrifuged at 2000xg for 10 minutes at 4°C. Serum was removed and stored at -80°C until testing by ELISA for 17-β estradiol or LC-MS/MS for progesterone.

### 2.6 ELISAs

Estrogen concentration in serum samples was measured using the Calbiotech E2 ELISA (ES180) according to the manufacturer’s instructions. IFNϵ concentration in vaginal swab samples was determined using the Biomatik (EKC37135) mouse IFNϵ ELISA. Absorption values were measured on a Turner Modulus instrument and data were analyzed with GraphPad Prism software.

### 2.7 LC-MS/MS determination of progesterone from serum

For each progesterone measurement, 100 ul of serum was vortexed with 10 uL of internal standard (100 ng/mL d9 labeled progesterone Sigma-Aldrich, St. Louis, MO, USA), then combined with 100 mL LC-MS grade water. The diluted sample was then deposited into a Biotage ISOLUTE SLE+ 400 µL 96-Well Plate and a vacuum pulse was applied to load the sample. Samples were allowed to interact with SLE+ packing for five minutes before elution with ethyl acetate (2x aliquots of 400 mL each). Final elution was achieved using vacuum and the resulting eluent was evaporated to dryness under nitrogen at 37°C using a TurboVap LV (Biotage, Uppsala, Sweden). The residue was reconstituted in 80 mL of LC-MS grade acetonitrile and then filtered using a 0.22μm filter tube (CoStar Group, Washington DC, USA). The filtered sample was then subject to LC-MS/MS analysis using a Shimadzu LCMS-IT-TOF system (Shimadzu Scientific, Kyoto, Japan). Separation occurred with a UCT C18 column (100 x 2.1 mm, 1.8-micron particle size) maintained at 50°C. The HPLC program included a gradient elution with 1mM ammonium fluoride in water (A) and acetonitrile (B) with a 30% B to 100% B ramp over 10 minutes. The mass spectrometer was maintained in +ESI mode using nitrogen as a nebulizing gas (1.5 L/min). Quantification was achieved using the direct MS channel for progesterone and d9-progesterone, m/z 315.01 and m/z 324.12, respectively. On each day of sample analysis, a five-point calibration curve (5, 10, 20, 50, and 100 ng/mL progesterone, Sigma-Aldrich, St. Louis, MO, USA) was prepared by spiking blank serum with progesterone stock solution to achieve the desired concentrations. Then the progesterone spiked samples were subject to the same internal standard concentration and SLE+ extraction prior to analysis. Calibration curves were created by plotting peak area ratio (progesterone: d9-progesterone) versus spiked progesterone concentration and a best-fit line was generated. The best-fit line was used to calculate progesterone concentration in study sample using their peak area ratios.

### 2.8 Tissue collection

Mock or *C. muridarum* infected mice were sacrificed on day 9, 10 or 21 pi. Genital tracts were observed for gross pathology *in situ.* The genital tracts were scored for redness and swelling as described in **Table 1**. Photos were taken to create a scoring reference sheet so that genital tract pathology was consistently scored between groups and experiments. Afterwards, the tissues were excised from the animals and separated into 3 parts: ovaries, uterine horns, and cervix. Tissues for flow cytometry and RNA analyses were minced and placed in 1mg/ml collagenase in Gibco Hank’s balanced salt solution (HBSS). After incubation in collagenase for 30 minutes at 37°C, tissues were homogenized with a pestle. A portion (100ul) of each sample was then mixed with RLT buffer (Qiagen RNeasy Kit) to be used for RNA analysis. The remainder of each sample was filtered and rinsed with MEM followed by 0.5 M EDTA. Single cells were collected by centrifugation and resuspended in MEM for flow cytometry analysis. This tissue collection method was derived from Nagarajan et al. (Nagarajan et al., 2012).

**Table 1:** Pathology score criteria.

Score	Explanation
**0**	No redness, swelling, or other pathology present
**1**	Swollen with no color OR pink with no swelling
**2**	Pink and mild swelling
**3**	Red with or without swelling
**4**	Dark red and severe swelling
**5**	Dark red, severe swelling, and hydrosalpinx or cyst(s)

### 2.9 Flow cytometry analysis

Tissues were collected as described above. Cells from the ovary, uterine horn, and cervix from each mouse were combined to ensure an ample number of cells for flow cytometric measurement. A blocking buffer including 5% BSA, rabbit serum, EDTA, and Fc blocking antibody (CD16/CD32 Rat anti-Mouse; BD Biosciences 553142) was added to each sample to block leukocyte staining. Cells were stained overnight with the following antibodies at 4°C: Pacific Blue™ Hamster Anti-Mouse CD3e (T cells), PE Rat Anti-Mouse CD19 (B cells), Rat Anti-Mouse Ly-6B.2 Alloantigen: FITC (PMNs), PE-Cy™7 Rat Anti-Cd11b (granulocytes), PerCP-Cy™5.5 Rat Anti-Mouse Ly-6G and Ly-6C (GR-1) (granulocytes), and AF 647 Rat Anti-Mouse F4/80 (monocytes/macrophages), or their isotype control antibodies. All antibodies were ordered from BD Biosciences, except for Ly-6B.2, which was ordered from BioRad. The following day, samples were washed with stain buffer, fixed in 4% paraformaldehyde, suspended in stain buffer, and analyzed using a BD Fortessa flow cytometer with FACSDiva software. Murine blood was collected from a cull mouse *via* cardiac puncture and stained with all antibodies for use as a positive control. Lymphocyte and myeloid cell populations were gated based on forward versus side scatter plots and positive antibody staining in a fully stained blood sample. Lymphocytes were gated based on positive CD3e versus CD19 staining and myeloid cells were gated based on positive Ly-6G and Ly-6C versus Ly-6B.2, and CD11b versus F4/80 staining.

### 2.10 TruSeq targeted RNA expression and RT-PCR analyses

RNA was collected as described above and processed as detailed in the TruSeq targeted RNA expression guide (Illumina). Briefly, the quality of RNA was confirmed using an Agilent Technologies RNA 6000 Nano Kit and 2100 Bioanalyzer. RNA from each sample was reverse transcribed to cDNA. A custom oligo pool with primers specific to our targeted genes (**Supplemental Table 1**) was used to amplify the cDNA sequences by PCR. A second limited cycle PCR was used to add sample specific index oligos to the amplified sequence libraries. Pooled libraries were quantified and prepared for sequencing. Library preparation was performed in the East Tennessee State University Molecular Biology Core Facility (RRID: SCR_021106). The library was analyzed by the University of Tennessee Genomics Core Facility (Knoxville, TN) on an Illumina MiSeq. Data was analyzed using Qiagen’s CLC Genomics Workbench following the RNAseq workflow. *C. muridarum* 16S rRNA (forward: 5’-aagfttttcttaacaatgcaaatgagatag-3’, reverse: 5’-ctgcagcctccgtagagtctgggcagtgtc-3’) and murine 18S rRNA (forward: 5’-ccggacacggacaggattga-3’, reverse: 5’-gcatgccagagtctcgttcg-3’) transcripts from infected WT and ERαKO genital tissues were amplified by RT-PCR (Invitrogen). The amplimers were ran on an ethidium bromide-stained agarose gel. Bands were visualized and quantified using a BioRad G-box system with Syngene analysis software.

### 2.11 Statistics

Unless otherwise stated the data presented represent data collected from three independent experiments with 4-8 mice/group. Data was analyzed using Microsoft Excel and GraphPad Prism software. Bacterial shedding between mouse groups was compared using two-way ANOVA and independent 2-sample t-tests where appropriate. Immune cell populations measured by flow cytometry were reported as percentages of the total number of cells measured, and compared using a one-way ANOVA and 2-sample t-tests. Qiagen’s CLCBio Genomics Workbench was used to analyze the TruSeq targeted RNA expression data. The differential expression for RNASeq tool using trimmed mean of M values (TMM) normalization was used to compare gene expression among groups. Groups with statistically significant fold changes were reported. In all analyses, significance was defined as p<0.05.

## 3 Results

### 3.1 Physiologically relevant levels of 17-β estradiol protects mice from *C. muridarum* infection

**Figure 1:**
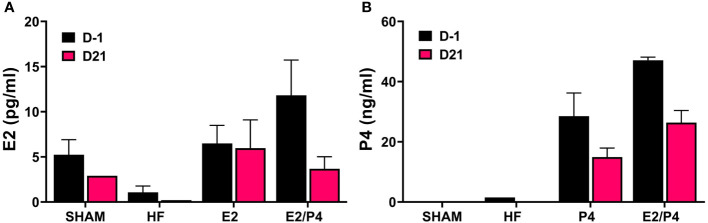
Measurement of E2 and P4 from serum. The concentration of 17-β estradiol **(A)** and progesterone **(B)** in mouse sera was determined by ELISA or LC MS/MS, respectively as described in the Methods. E2: 17-β estradiol, P4: progesterone, E2/P4: combination of 17-β estradiol and progesterone, SHAM: sera from surgical sham mice treated with DMPA. Data shown represent the average hormone concentration ± standard deviation in pooled sera from 16 mice collected across 2 independent experiments.

The murine model is the most commonly used whole animal model in chlamydial research. Thus, we chose to use an ovariectomized (OVT) mouse model to examine the effects of long-term exposure to physiologically relevant concentrations of female sex hormones on chlamydial infection. We also chose to use a chlamydial inoculum size that is consistent with the amount of EB shed from female patients to further mimic a realistic infection environment (Eckert et al., 2000). Silastic capsules containing either sesame oil (SO) only (HF), 17-β estradiol in sesame oil (E2), progesterone in SO (P4), or a combination of 17-β estradiol and progesterone in SO (E2/P4) were inserted subcutaneously into OVT BALB/c mice. Mice with a sham ovariectomy and sham capsule surgery were also included in the study as controls. To ensure the hormone levels were within physiological range, serum concentrations of 17-β estradiol and progesterone were measured in serum collected the day before infection (day -1) and at day 21 post infection (pi, **Figure 1**). The data confirm that physiologically relevant concentrations of estrogen and/or progesterone were obtained in the E2, P4 or E2/P4 groups prior to and maintained throughout chlamydial infection compared to HF control animals. Interestingly, DMPA-treated SHAM animals also expressed endogenous E2, but not P4 (**Figures 1A, B**).

**Figure 2:**
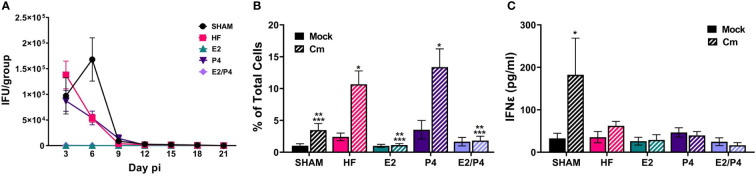
*Sex hormones alter* bacterial vaginal shedding and immune response *to C muridarum* (Cm) in infected ovariectomized mice. **(A)**. EB shedding was monitored by chlamydial titer assays from *C muridarum* infected, hormone-treated mice for 21 days pi. Data shown represent the average (n= 12-29 mice/group/day) IFU/group ± SEM. **(B)** Flow cytometry analysis was performed to detect CD3 positive T cells in the genital tracts of mock- or Cm infected, hormone-treated OVT mice at day 10pi. Data shown represent the average (n= 12 mice/group) percentage of total cells measured ± SEM. Asterisks indicate significant differences (P ≤ 0.05). *Cm vs mock, **HF (Cm), ***P4 (Cm), vs experimental group. **(C)** The concentration of IFNϵ was detected in vaginal swab samples on day 3pi by ELISA. Data shown represent the average (n= 7 mice/group) IFNϵ concentration ± SEM. Asterisks (*) indicate significant differences between Cm and mock infection (P ≤ 0.05).

At day 0 HF, E2, P4, E2/P4 or sham mice were mock or *C. muridarum* vaginally infected. Vaginal EB shedding was monitored for 21 days following infection. There was no significant difference in EB shedding between sham, HF, or P4 exposed mice. On the contrary, estrogen exposure, alone (E2) or in combination with progesterone (E2/P4) abolished EB shedding, indicating that 17-β estradiol protected the mice from establishing chlamydial infection (**Figure 2A**).

### 3.2 Natural and synthetic hormones influence the immune response to *C. muridarum*

Given that studies have shown that hormones influence immune cell function and signaling, we sought to determine if the observed changes in EB shedding in OVT mice are due to hormonal influences on the immune response. A subset of hormone-exposed OVT and sham mice were sacrificed on day 10pi to determine the immune cell phenotype present in mock or *C. muridarum* (Cm)-infected genital tracts at that time (**Figure 2B**; **Supplemental Figure 2**, **3**). We chose this time as it represented the initial decrease in EB shedding from infected mice. Additionally, other studies have reported that *C. muridarum* ascends the genital tract and the immune response to *Chlamydia* has been established by 6-10 days post infection (Darville and Hiltke, 2010; Frazer et al., 2013; Campbell et al., 2014; Poston et al., 2017; Murthy et al., 2018). Flow cytometry analysis indicated that the hormonal environment did not significantly impact recruitment of PMNs, monocytes/macrophages or B cells to the infected genital tract (**Supplemental Figure 3**). However, the T cell population present during chlamydial infection was altered by the hormonal conditions. There were significantly fewer T cells present in E2 and E2/P4 groups compared to sham, HF, and P4-treated groups (**Figure 2B**). These data are unsurprising, given the lack of EB shedding in the E2- and E2/P4- exposed mice. Combined with the results from chlamydial titer analysis, the increased T cell presence in the HF- and P4- treated mice suggest that T cells are being recruited to the genital tract in these hormonal conditions in response to *C. muridarum* infection. Interestingly, sham mice, which were treated with DMPA, a synthetic progestin, also had significantly fewer numbers of T cells on day 10pi compared to the HF and P4 groups (**Figure 2B**). These data suggest that DMPA elicits different effects on chlamydial pathogenesis and may blunt the immune response to *C. muridarum.*

Fung, et al., previoulsy reported that estrogen regulates expression of the type-I interferon, Interferon-ϵ (IFNϵ) in mice (Fung et al., 2013). Furthermore, infected Interferon-ϵ knock-out (KO) mice shed more *C. muridarum* EBs than wild-type mice (Fung et al., 2013). Thus we examined IFNϵ expression in our hormone-treated, mock or *C. muridarum* infected, OVT mice. Exspoure to E2 or P4 alone or in combination did not significantly elevate IFNϵ expression on day 3pi compared other hormonal exposure conditions in either mock or infected mice. Interestingly, DMPA-exposed, *C. muridarum*-infected Sham mice expressed significantly more IFNϵ compared to mock or infected HF, P4, E2 or E2/P4 exposed mice (**Figure 2C**). These data indicate that IFNϵ is not responsible for preventing *C.muridarum* infection in E2 and E2/P4 exposed mice compared to HF, P4 or Sham animals (**Figure 2A**). Additionally, these data indicate that DMPA differenitally regulates expression of IFNϵ during chlamydial infection compared to naturally occuring sex hormones in BALB/c mice.

### 3.3 The absence of ERα in mice alters *C. muridarum* vaginal shedding

**Figure 3:**
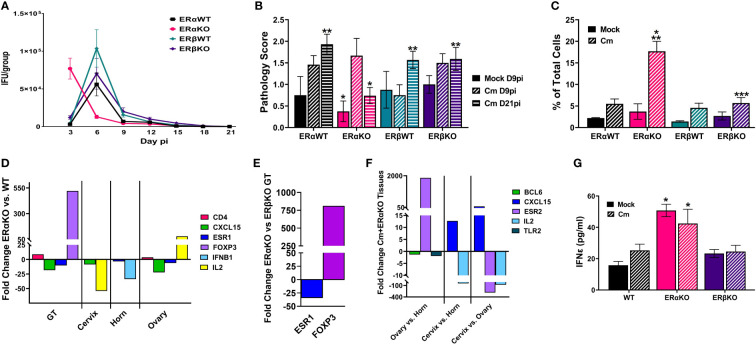
Estrogen receptors alter bacterial shedding, pathology, and the T cell response *to C muridarum* (Cm) in infected C57Bl/6 mice. **(A)**. EB shedding was monitored by chlamydial titer assays from *C muridarum* infected C57Bl/6 WT, ERαKO or ERβKO mice for 21 days pi. Data shown represent the average (n= 24 mice/group/day) IFU/group ± SEM. **(B)**. Gross genital tract pathology was assessed using the scoring criteria in **Table 1** on day 9 or 21pi. Data shown represent the average pathology score ± SEM/group. (n=4-21mice/group/day) An asterisk (*) indicates significant difference from Cm-ERαKO D9pi, **Significantly different from Cm-ERαKO D21pi. **(C)** Flow cytometry analysis was performed to detect CD3 positive T cells in the genital tracts of mock or Cm-infected, hormone-treated OVT mice at day 9pi. Data shown represent the average (n=12 mice/group) percentage of total cells measured ± SEM. Asterisks indicate significant differences (P ≤ 0.05). *Cm vs mock, **ERαWT Cm vs Cm group, ***ERβWT Cm vs Cm group. **(D-F)**. Fold changes in expression of genes determined to be significantly different using the CLC Bio Genomics Workbench RNAseq deferential expression analysis tool. **(D)**. Comparisons between Cm-infected ERαKO and WT whole genital tracts (GT), or cervix, horn and ovary tissues. **(E)**. Comparisons between Cm-infected ERαKO and ERβKO GTs. **(F)**. Comparisons between Cm-infected ERαKO cervix, horn and ovary tissues. **(G)**. The concentration of IFNϵ detected in vaginal swab samples on day 3pi by ELISA. Data shown represent the average (n= 4-9 mice/group) IFNϵ concentration ± SEM. Asterisks (*) indicate significant differences between Cm and mock infection (P ≤ 0.05).

Previous studies have shown that estrogen receptors are expressed differently in various cell types and tissues (Eyster, 2016) and that signaling *via* ERs alter immune cell function and chlamydial infection *in vitro* (Hall et al., 2011). Thus, we wanted to examine the specific effects of ERα and ERβ on chlamydial infection *in vivo*. ERα and ERβ KO mice, along with their respective C57Bl/6 WT strains were vaginally infected with *C. muridarum.* Bacterial shedding was monitored for 21 days. ERαWT and ERβWT mice shed EB in a similar pattern, with a peak in EB shedding on day 6pi that decreased by day 9pi (**Figure 3A**). ERβKO mice had a similar shed curve to the WT mice, apart from increased, although not statistically significant (p= 0.99), EB shedding on day 6pi. Conversely, peak EB shedding from infected ERαKO mice occurred on day 3pi with no EB collected by day 15 pi. Indeed, significantly more EB were shed in the ERαKO mice on day 3pi compared to the WT or ERβKO animals. These data indicate that in the presence of only ERβ (ERαKO), chlamydial infection progressed faster and was cleared more quickly from the genital tract compared to WT animals expressing both ERs or animals expressing only ERα.

### 3.4 ERα and ERβ influence the pathology of the genital tract after chlamydial infection

At the time of sacrifice, genital tracts from all mice were examined *in situ* for gross pathology and assigned a score based on redness and swelling (**Table 1**). On day 9pi, genital tracts from infected WT or ERKO mice were collected and accessed for the presence of *C. muridarum* 16S rRNA transcripts by rtPCR. As shown in **Supplemental Figure 4**, chlamydial transcripts were present in the cervix, uterine horn, and ovary of both WT and ERαKO mice, indicating the presence of ascending infection. the genital tracts of all *C. muridarum* infected mice exhibited increased redness and swelling compared to the mock infected groups, with ERαKO mice having the greatest pathology on day 9pi (**Figure 3B**). By day 21pi, the gross pathology exhibited in infected ERαKO mice had decreased, whereas pathology in the WT or ERβKO mice continued to be elevated throughout the experiment. The ERαKO mice on day 21pi had significantly less redness and swelling than the WT and ERβKO mice (**Figure 3B**). These data are consistent with EB shedding from the WT and ERKO animals, correlating with the rapid clearance of infection by ERαKO mice.

### 3.5 The absence of estrogen receptors alters the immune response to *C. muridarum* infection *in vivo*

To examine the immune cell response to *C. muridarum* infection in C57BL/6 WT and ERKO mice, we collected genital tracts from mock and infected WT and ERKO mice on day 9pi. We used flow cytometry to examine the proportion of myeloid and lymphocyte immune cells present in genital tract at that time (**Figure 3C**; **Supplemental Figures 5**, **6**). At day 9pi, ERαKO mice infected with *C. muridarum* had significantly more T cells present in the genital tract than infected ERβKO and WT mice. ERαKO infected mice also had significantly more T cells present than their corresponding mock-infected group (**Figure 3C**). Although the data are limited to one time during infection, these data suggest that ERα is involved in the regulation of the T cell response to chlamydial infection in mice.

To further investigate the immune response to *C. muridarum* infection, we examined the expression of 19 immune marker genes (**Supplemental Table 1**) in mock and infected WT and ERKO tissues collected at day 9pi using Illumina TruSeq analysis. Gene expression was compared in whole genital tract (GT) tissues as well as in separated tissues for the cervix, horn, and ovary. *C. muridarum*-infected ERαKO mice had upregulated *CD4* and *FOXP3* expression compared to infected WT mice, indicating that the absence of ERα resulted in increased helper T cells (**Figure 3D**). Expression of *FOXP3* was also significantly increased in the GTs of infected ERαKO mice compared to infected ERβKO mice (**Figure 3E**), suggesting that the observed increase in T cells in ERαKO mice is due to increased T regulatory cells (Tregs). Conversely, we observed downregulated levels of *CXCL15* and *ESR1* expression in ERαKO verses WT infected GTs (**Figure 3D**). *ESR1* encodes ERα, thus the downregulation of this gene was expected in ERαKO tissues. *ESR1* was also significantly decreased in both the horn and ovary of infected ERαKO verses infected WT mice. In addition to the whole GT, expression of *CXCL15*, which encodes IL8 in mice, was decreased in the cervix and ovary of ERαKO mice compared to WT. *IL2* expression was differentially altered, being increased in the ovary and decreased in the cervix of ERαKO compared to WT. Cytokine expression in the horn of ERαKO mice was decreased with significantly less *IFNB1* expression than WT mice. Lastly, the ovaries of ERαKO mice had significantly more *CD4* expression than the WT mice, suggesting that more CD4+ T cells were present.

We also observed changes in gene expression in the upper and lower genital tract in the ERαKO tissues (cervix, horn, and ovary). There was upregulation of *CXCL15* and downregulation of *IL2* in the cervix compared to the horn and ovary in infected ERαKO mice. Expression of ERβ (*ESR2*) was upregulated in the ovary of the ERαKO mice as expected, while expression of *BCL6* and *TLR2* was downregulated compared to the horn (**Figure 3F**).

Lastly, we examined secretion of IFNϵ from WT (C57Bl/6) or ERKO mice during mock or chlamydial infection. On day 3pi there was significantly more IFNϵ expressed in ERαKO animals in both the mock and *C. muridarum*-infected groups compared to WT or ERβKO animals (**Figure 3G**). These data indicate that IFNϵ is constitutively expressed in ERαKO animals and may play a role in the more rapid clearance of *C. muridarum* infection in ERαKO mice compared to WT or ERβKO mice. Together, these data support the observation that estrogen receptors play a role in the immune response to chlamydial infection in mice throughout the entire genital tract.

## 4 Discussion and conclusions

Previous studies from our lab demonstrated that 17-β estradiol (E2) treatment enhanced *C. trachomatis* infection in an IK/SHT290 co-culture model, and that progesterone (P4) antagonized the enhancement observed with E2-only exposure (Kintner et al., 2015). Further, E2 was found to increase attachment of *C. trachomatis* EB to human epithelial cells, while P4 reduced attachment (Maslow et al., 1988). Based on these data, we sought to examine the effects of E2 and P4 on chlamydial infection *in vivo.*

There are several reports that E2 and P4 treatment affect rats and mice differently than humans, swine, and guinea pigs. Treatment of animals with E2 has various effects on chlamydial infection. *C. caviae* is a species of *Chlamydia* that causes inclusion conjunctivitis in guinea pigs, known as guinea pig inclusion conjunctivitis (GPIC). E2 treatment (1mg 17β-estradiol injection day for 14 days prior to infection) of guinea pigs prior to *C. caviae* infection resulted in a longer lasting infection with higher numbers of EB collected from vaginal swabs, as well as more pathology than non-E2 treated guinea pigs (Rank et al., 1982). Further, genital epithelial cells isolated from swine exhibited greater chlamydial attachment and infectivity when the cells were collected during the estrogen-dominant phase of the animal’s estrus cycle (Guseva et al., 2003). Conversely, treatment of rats with E2 prior to chlamydial infection (10ug 17β-estradiol injection day for 3 days prior to infection) results in the complete protection from infection, and these animals require P4 treatment for successful infection of the genital tract (2000; Kaushic et al., 1998). Likewise, Pal, et al, demonstrated that mice were more susceptible to *C. trachomatis* infection during diestrus compared to estrus (Pal et al., 1998). These studies illuminate the varied actions of sex hormones on chlamydial infection *in vivo* depending upon the experimental model, including differences the chlamydial species/serovars and animal hosts examined.

Additionally, the hormonal environment evaluated in these studies varied. In some cases, susceptibility to infection is examined across the estrus cycle without hormone supplementation, whereas in others animals are exposed to exogenous hormones. Administration of hormones in these studies also varied in their delivery and dosages, as noted in the examples above. Hormones were usually delivered *via* multiple injections or intravaginal administration, occurring over several days prior to infection. The dosage of estradiol used in previous investigations varied from 10ug- 5mg/day depending on the animal species and study (Rank et al., 1982; Pasley et al., 1985; de Jonge et al., 2011; Quispe Calla et al., 2021). Importantly, serum concentrations of estrogen and/or progesterone were often not measured to determine if a physiologically relevant concentration was achieved. In fact, only one of the referenced studies measured serum hormone concentrations of E2 following injection of the hormone and reported high levels of E2 in the exposed animals (Rank et al., 1982).

The current study aimed to explore the interactions between female sex hormones, estrogen receptors, *C. muridarum* and host immunity using two murine models of chlamydial infection, estrogen receptor knockout mouse strains and hormone-exposed OVT mice. Using the silastic capsule method of hormone administration, we were able to introduce continuously-released, physiologically relevant levels of E2 and P4 to OVT mice in order to study hormone-specific features of chlamydial pathogenesis and host immunity. We found that physiologically relevant levels of E2, even in the presence of P4, completely protected mice from establishing *C. muridarum* infection. A previous study infecting E2-, P4-, or combination E2/P4-treated OVT rats with *C. muridarum* reported that E2 completely protected the rats from chlamydial shedding and inflammation while P4 treatment resulted in greater than 10^9^ IFU/ml detected in vaginal washes and severe inflammation in the genital tracts. However, this study found that combination E2/P4 treatment of these rats prior to chlamydial infection did not protect the rats from infection, measured by chlamydial shedding, although the combination-treated rats did have reduced inflammation compared to the P4-treated rats (Kaushic et al., 2000). Thus, our results reiterate the protective effect of estrogen on chlamydial infection in mice even when progesterone is present These results, along with the studies referenced above, demonstrate that the effects of estrogen and progesterone in rodents is opposite to that observed in human cells or other animal models of chlamydial infection. However, it is known that sex hormone signaling in human endometrial epithelial and/or stromal cells influences chlamydial infection (Hall et al., 2011; Kintner et al., 2015). It is possible that estrogen acts directly or indirectly upon murine epithelial cells to prevent chlamydial infections from establishing. This experimental model also provides a system of long-term hormone exposure in which to expand future investigations on the effect of individual hormones on chlamydial infection.

The short estrus cycle in mice prevents genital *Chlamydia* infection from being established in actively cycling mice. Progesterone in the form of depo-medroxyprogesterone acetate (DMPA, Depo-Provera) is widely used in the field of chlamydial research as a pretreatment to stop the estrus cycle before chlamydial infection by causing mice to be suspended in the diestrus phase (Kaushic et al., 2003). Therefore, the existing literature highlights the necessity of progesterone treatment in the form of DMPA to establish chlamydial infection in mice. This model, however, does not allow for investigation of the effects that naturally occurring progesterone- or estrogen-dominant environments have on chlamydial infection in mice. DMPA treatment of mice has previously been shown to reduce antibody response to HSV-2 following immunization compared to P4 treatment (Kaushic et al., 2003). Likewise, Armitage, et al., demonstrated that the polymeric immunoglobulin receptor (PlgR) is up-regulated by estrogen and chlamydial infection in mice, whereas DMPA down-regulates *plgR* expression (Armitage et al., 2017). As PlgR transports dimeric IgA to musocal surfaces, DMPA treatment may decrease mucosal immunity to chlamydial infection. Our current study demonstrates that DMPA-treated mice had reduced T cells present on day 10pi compared to HF or P4-treated OVT mice despite equally robust bacterial shedding early during infection. Additionally, we observed a significant increase in INFϵ expression in the *C. muridarum*-infected, Sham, DMPA-treated BALB/c mice compared to the other hormonal conditions examined. These findings support the concept that DMPA influences the immune response to infection differently than endogenous progesterone. DMPA may suppress the immune response to infection in the female genital tract, which highlights the need for a model of chlamydial infection that does not require pretreatment with DMPA.

Chlamydial infection in ERαKO mice resulted in a quicker progression and clearance of *C. muridarum* infection compared to WT or ERβKO mice. These observations indicate that ERα and ERβ specific signaling events impact chlamydial infection, as ERαKO mice still express ERβ. The effects of ER signaling on chlamydial infection could occur at the level of direct epithelial cell signaling, hormone-stimulated stromal cell signals, or alterations of the immune response. We found that T-cells, *FOXP3* and IFNϵ expression were significantly increased in chlamydia-infected ERαKO compared to WT or ERβKO mice. Although our data are limited to a snapshot of time during the infection process, these data suggest that ER-stimulated alterations in the immune response contribute to observed changes in chlamydial infection.

Estrogen receptor signaling can either enhance or dampen innate immune cell function. Estrogen signaling has been shown to increase IFNγ and FOXP3 while dampening IL2 and CXCL-15 expression (McMurray et al., 2001; Nakaya et al., 2006; Tai et al., 2008; Mircheff et al., 2010; Moore-Connors et al., 2013). Expression of the type I interferon, IFNϵ, is specific to the female genital tract. Its expression is regulated during the menstrual/estrus cycle with estrogen stimulating the highest level of expression (Marks et al., 2019). Interestingly, we did not observe increased release of IFNϵ in E2-exposed OVT mice as was reported by Fung, et al. (Fung et al., 2013). This observation is likely due to differences in our experimental design, specifically our hormone-delivery system and E2 concentrations. It is also possible that there are differences in BALB/c versus C57Bl/6 mouse strains. We observed significantly higher expression of IFNϵ in ERαKO mice compared to C57Bl/6 WT or ERβKO mice. This observation was repeated in both mock and *C. muridarum*-infected animals, indicating that the absence of ERα was responsible for elevating IFNϵ. Fung, et al., also noted that IFNϵ expression inhibited chlamydial infection (Fung et al., 2013). These observations raise the question of whether IFNϵ expression is upregulated in ERαKO mice *via* ERβ signaling and suggest that specific ER signaling can influence chlamydial infection *via* IFNϵ expression.

Despite being similar in structure and in function, ERα and ERβ are located on different chromosomes and are expressed differentially on various tissues throughout the body. In mice and in humans, ERα is highly expressed in the ovaries as well as the uterus. Conversely, ERβ is the dominant ER in the ovaries, but is not expressed in the uterus (Wang et al., 2000; Jia et al., 2015; Hamilton et al., 2017). Analysis of gene expression in *C. muridarum-*infected ERαKO mice indicate that ERβ was not upregulated in the horn to compensate for the loss of ERα. Additionally, ERs are expressed differentially on immune cells. In humans, ERα and ERβ are expressed on most immune cells at varying levels (Kovats, 2015). ERα has been reported to have higher expression on CD4+ T cells compared to ERβ, while ERβ was more highly expressed on B cells. The same study reported no differences in ER expression in Th1 and Th2 cells (Phiel et al., 2005). In mice, ERα but not ERβ was detected in macrophages, CD4+ and CD8+ T cells (Lambert et al., 2005). Furthermore, deficiency of ERα expression increased CD4^+^ T cell stimulation in murine macrophages (Lambert et al., 2005). This observation suggests that the faster clearance of *C. muridarum* infection in ERαKO mice is directly related to the increased production of T cells under ERα deficient conditions compared to WT ER expression or ERβ deficiency.

Our finding that ERαKO mice had a significant increase in T cells and *FOXP3* expression during *C. muridarum* infection suggests that T regulatory cells (Tregs) are involved in chlamydial clearance under ERα deficient conditions. In fact, Treg production is known to be increased in the presence of *C. muridarum* infection *in vitro* and *in vivo*, possibly aiding the clearance of chlamydial infection *via* stimulation of Th17 production [44]. Studies have shown that estrogen signaling *via* either ERα or ERβ can stimulate Treg production. E2 exposure elevated *FOXP3* expression and Treg production in WT mice compared to ERαKO mice, suggesting that ERα signaling was involved (Polanczyk et al., 2004). Conversely, Xiong, et al., demonstrated that ERβ signaling is required for *FOXP3* expression and Treg production in response to respiratory tract infections (Xiong et al., 2021). Given these observations, our study suggests that ER signaling differentially alters the progression of *in vivo C. muridarum* infection *via* regulation of the T cell response.

Together, our data indicate that E2 and ERs have important roles in the pathogenesis of *C. muridarum* infection in mice. While we have focused on the contributions of hormone-induced immune responses to chlamydial pathogenesis, another possibility is that ER signaling in infected epithelial cells contributes to the observed alterations in chlamydial infection as has been demonstrated *in vitro* (Kovats, 2015). Additionally, our studies examined the role of individual ERs on chlamydial infection. Future studies, investigating chlamydial infection in a double ERα/ERβ knockout murine model or by treating ERαKO mice with an ER antagonist would provide a better understanding of the role ERs play in regulating chlamydial pathogenesis. Overall, these data reiterate the importance of hormone-stimulated signaling on chlamydial infection and highlight the need for future investigations to deepen our understanding about the ways sex hormones influence STI pathogens.

## Data availability statement

The data presented in the study are deposited in the NCBI SRA database repository, accession number PRJNA866545, https://www.ncbi.nlm.nih.gov/sra/PRJNA866545.

## Ethics statement

The animal study was reviewed and approved by East Tennessee State University, University Committee on Animal Care.

## Author contributions

AG, JK, SB, AC, BK, and JH designed and performed the experiments and analyzed the data. AG, SB, and JH wrote and edited the manuscript. All authors contributed to the article and approved the submitted version.
