# Supplementary material for: The hormonal environment and estrogen receptor signaling alters Chlamydia muridarum infection in vivo
Source: Front Cell Infect Microbiol. 2022 Dec 27;12:939944. doi: 10.3389/fcimb.2022.939944 (PMC9831676; doi:10.3389/fcimb.2022.939944)
Supplement: Supplementary file 1 [file DataSheet_1.docx]

**Supplemental Materials**

**
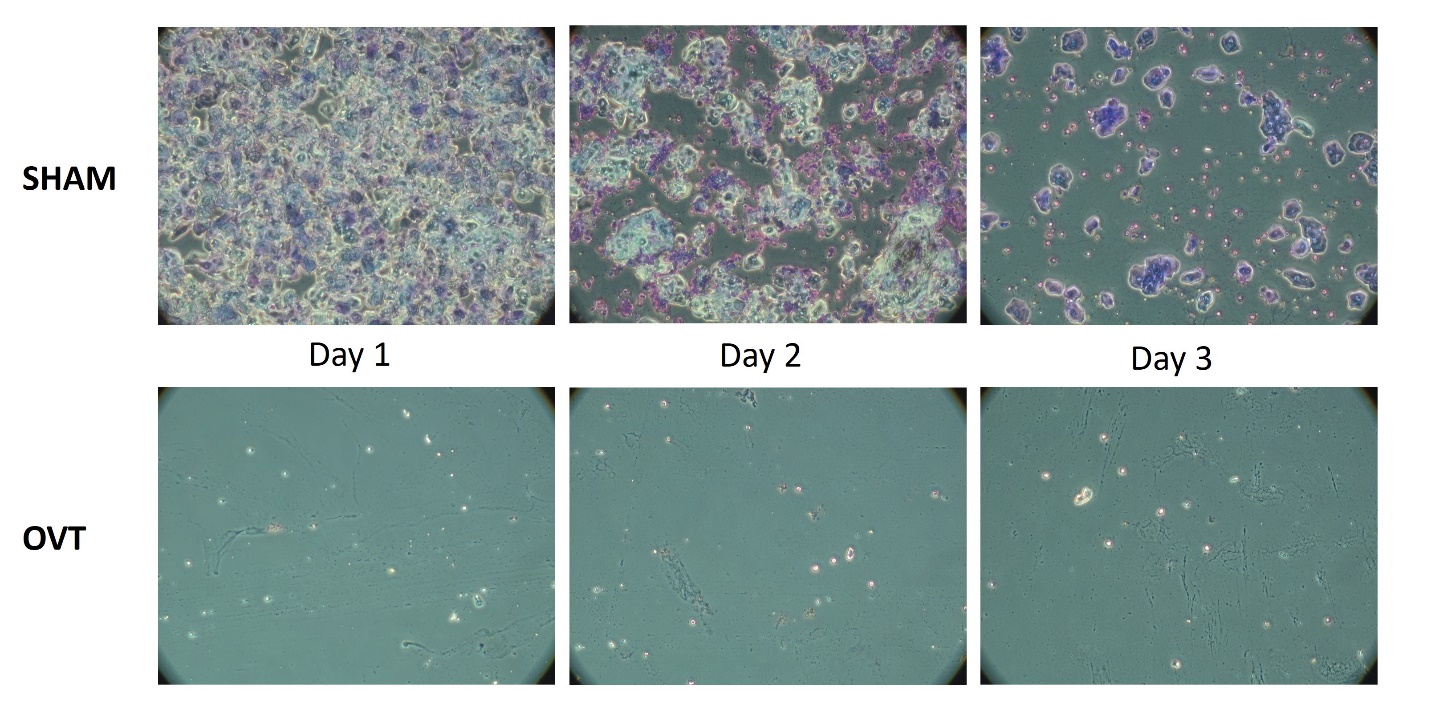
**

**Supplemental Figure 1:** Estrous cycling in SHAM vs OVT mice. Representative images of vaginal washes collected from SHAM or OVT surgical groups. Mice in the SHAM group continued to cycle as evidenced by the changes in shed cell types from cornified epithelial cells to nucleated epithelia and leukocytes compared to the OVT group in which few cells were collected on any day.

| **Supplemental Table 1: TruSeq Targeted RNA Expression Genes of Interest** | |
| --- | --- |
| **Gene Name** | **Abbreviation** |
| BCL6 Transcription Repressor | *BCL6* |
| Cluster of Differentiation 4 | *CD4* |
| Cluster of Differentiation 8 | *CD8* |
| C-X-C motif ligand15 | *CXCL15* |
| EGF-like module-containing mucin-like hormone receptor-like 1 | *EMR1 (F4/80)* |
| Estrogen receptor alpha | *ESR1* |
| Estrogen receptor beta | *ESR2* |
| Forkhead box P3 | *FOXP3* |
| Interferon alpha 1 | *IFNA1* |
| Interferon beta | *IFNB1* |
| Interferon gamma | *IFNG* |
| Interleukin-17a | *IL17A* |
| Interleukin 2 | *IL2* |
| Interleukin 4 | *IL4* |
| Transforming growth factor beta 1 | *TGFB1* |
| Toll-like receptor 2 | *TLR2* |
| Toll-like receptor 4 | *TLR4* |
| Tumor necrosis factor | *TNF* |


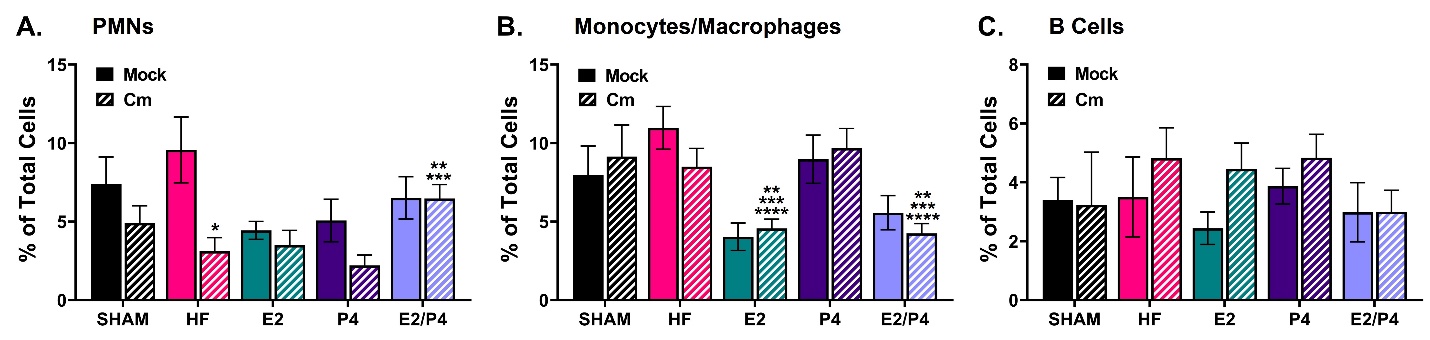


**Supplemental Figure 2:** Flow cytometry analysis of PMNs, Monocytes/Macrophages and B-cells in mock or Cm infected, hormone-exposed mice at 10pi. Data shown represent the average (n= 12 mice/group) percentage of total cells measured ± SEM. Asterisks indicate significant differences (P≤0.05). * Cm vs mock, ** HF (Cm), ***P4 (Cm), **** Sham (Cm) vs experimental group.


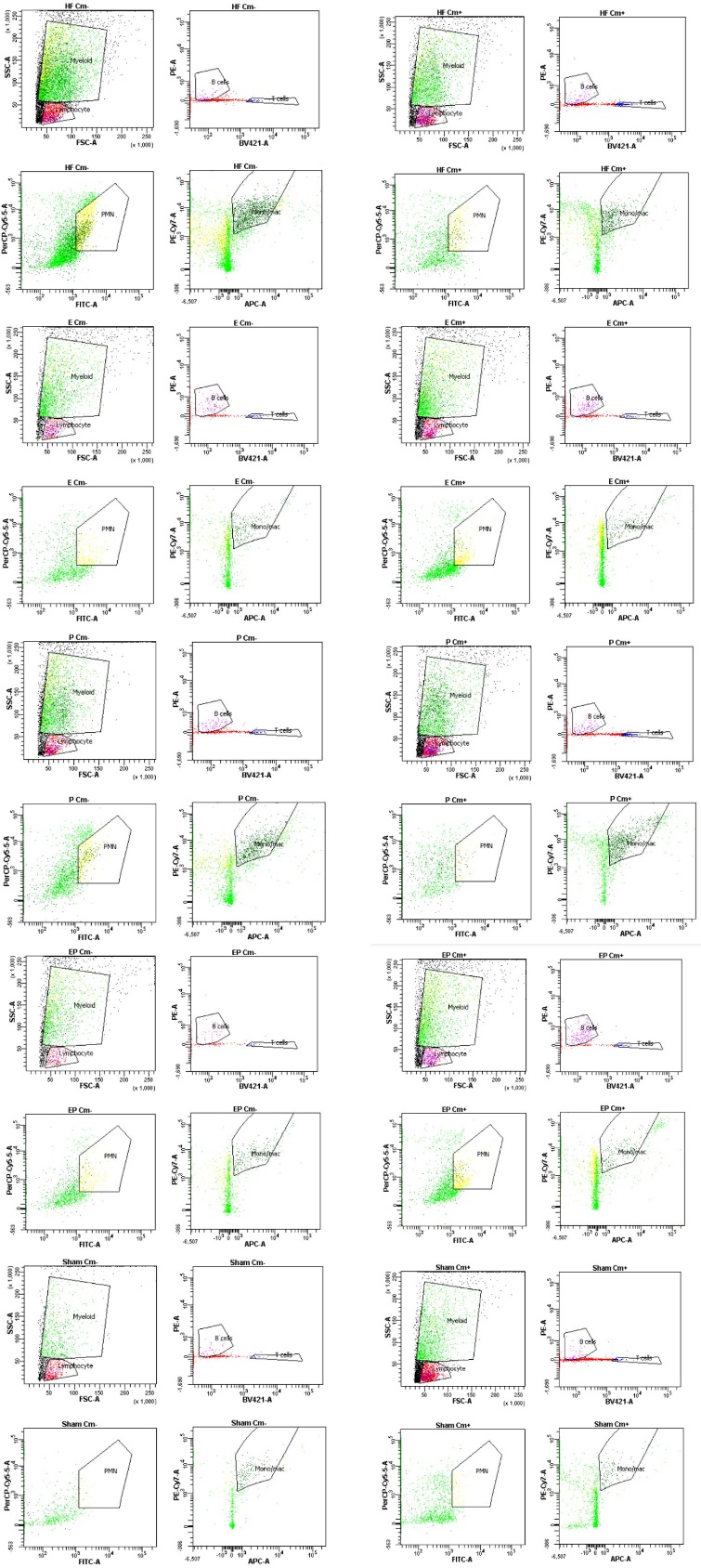


**Supplemental Figure 3:** Representative images of flow cytometry data collected from Mock (Cm-) or *C. muridarum* (Cm+) infected mice from each experimental group (HF, E2, P4, E2/P4, SHAM).

**Supplemental Figure 4**: Reverse transcription-PCR of *C. muridarum* 16S rRNA and murine 18S rRNA transcripts in the cervix, uterine horn or ovary of infected WT or ERαKO mice on day 9pi.


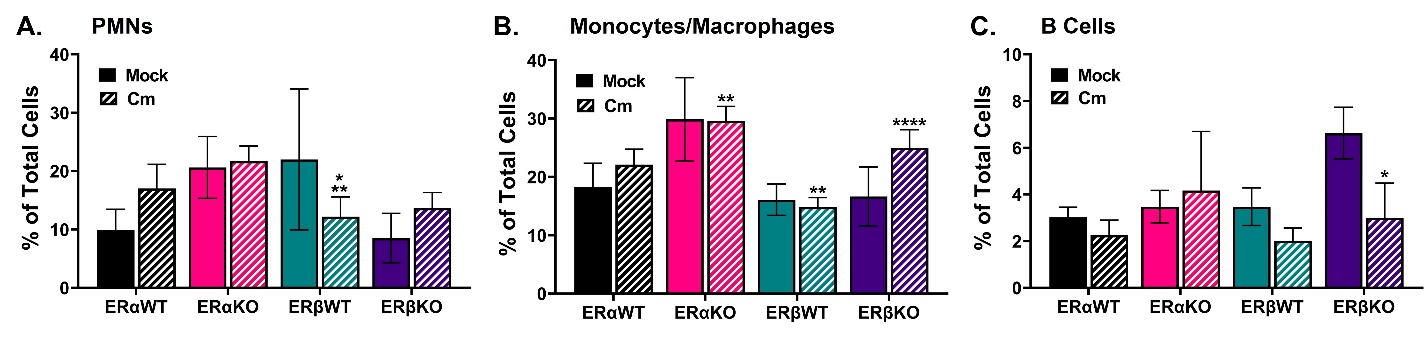


**Supplemental Figure 5:** Flow cytometry analysis of PMNs, Monocytes/Macrophages and B-cells in mock or Cm infected, ERαWT, ERαKO, ERβWT or ERβKO mice at 9pi. Data shown represent the average (n= 12 mice/group) percentage of total cells measured ± SEM. Asterisks indicate significant differences (P≤0.05). * Mock vs. Cm infection, ** Significant difference vs. ERαWT Cm, *** Significant difference vs. ERβWT Cm, **** Significant difference vs. ERβWT mock.


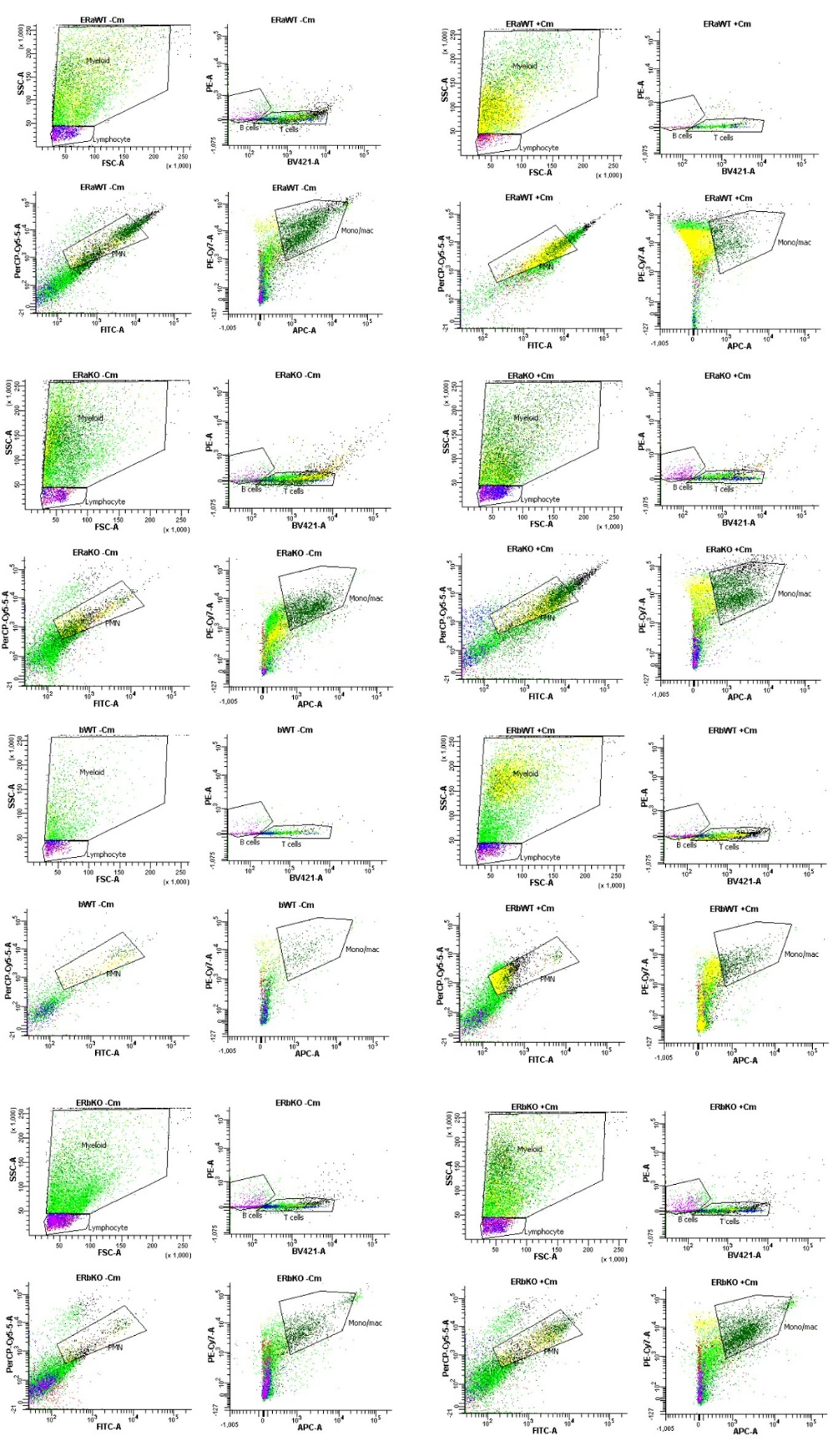


**Supplemental Figure 6:** Representative images of flow cytometry data collected from Mock (Cm-) or *C. muridarum* (Cm+) infected ERαWT, ERαKO, ERβWT or ERβKO mice.
